# Population-Level Associations of Diabetes Prevalence and Childhood Overweight with Kidney Cancer Mortality in Türkiye

**DOI:** 10.3390/jcm15197351

**Published:** 2026-09-22

**Authors:** Yasin Öztürk, Muammer Bilici, Kadir Yılmaz

**Affiliations:** 1 Department of Nephrology, Bülent Ecevit University, 67100 Zonguldak, Türkiye; 2 Internal Medicine, Bülent Ecevit University, 67100 Zonguldak, Türkiye; 3 Department of Statistics, Istanbul Commerce University, 34445 İstanbul, Türkiye; dekartezyen@hotmail.com

**Keywords:** kidney cancer, diabetes, childhood overweight, ecological analysis, mortality

## Abstract

**Background/Objectives**: This study aimed to conduct a population-level multivariate analysis of the associations of childhood overweight with kidney cancer mortality and diabetes. **Methods:** The study used data from the World Health Organization (WHO) and the World Bank for Türkiye between 2000 and 2023. Kidney cancer mortality and diabetes prevalence data were used from the WHO database. Health expenditure, life expectancy, mortality, and childhood mortality data were used from the World Bank database. **Results:** Spearman’s rho correlation analysis results showed that kidney cancer mortality was significantly correlated with health expenditure (r = −0.614; *p* < 0.05) and life expectancy (r = 0.596; *p* < 0.05). Generalized linear model results showed that associations of diabetes prevalence (B = 0.039; *p* < 0.01), health expenditure (B = −0.154; *p* < 0.01) and life expectancy (B = 0.061; *p* < 0.01) with kidney cancer mortality were statistically significant at the univariate level. However, associations of diabetes prevalence, childhood overweight and health expenditure with kidney cancer mortality at the multivariate level were statistically insignificant (*p* > 0.05). **Conclusions:** Although the prevalence of childhood overweight and diabetes was significant in univariate analysis, these associations were not significant in multivariate analysis. Therefore, we believe that the association of childhood overweight and diabetes with kidney cancer-related deaths must be further studied with confounders.

## 1. Introduction

Kidney cancer accounts for 3.8% of all new cancer cases [1] and is responsible for 175,000 deaths worldwide every year [2]. Despite the increasing availability of treatment methods, the high mortality rate indicates the need for further investigation of risk factors and prognostic factors. Risk factors for kidney cancer include the patient’s sociodemographic characteristics [3,4,5], genetic predisposition [6], hypertension [7], smoking [8], obesity [9], and diabetes [10]. Among these, diabetes and obesity are two health problems that are closely related to individuals’ nutrition and metabolism and trigger each other. In other words, diabetes can be considered a consequence of obesity and a mechanism of action on diseases.

Research on diabetes and kidney function reports that in diabetes, nutrition and impaired metabolism, along with chronic medications, have an association with kidney function [11,12,13]. In addition, studies linking kidney cancer and diabetes have reported obesity as both a risk factor for the development of kidney cancer and a significant factor in worsening the prognosis of the disease in patients [14]. A comprehensive study of 14,492 biopsy results reported that obesity is a significant risk factor for kidney cancer and is associated with mortality. Studies on diabetes and kidney cancer have also reported an increase in the prevalence and prognosis of kidney cancer in individuals with diabetes [15]. Studies on the relationships between diabetes and lifestyle, care, and stress, as well as studies on obesity and kidney disease, reveal the potential for a link between these diseases [16,17]. Additionally, the relationship between obesity and diabetes and kidney disease, and the relationship between diabetes and obesity, may point to a mechanism linking childhood overweight to diabetes and kidney disease. The conclusion drawn from all these clinical studies and literature reviews is that there may be a relationship between diabetes and childhood overweight and kidney cancer mortality. However, studies examining the associations of both risk factors are generally univariate and have analyzed diabetes either as a demographic factor or through intergroup differences. We did not find enough studies that examined the associations of diabetes and childhood overweight on kidney cancer mortality in a multivariate manner, focusing on causality rather than differences, using a correlational screening model. Such a study could better understand and elucidate the associations of diabetes and childhood overweight on kidney cancer mortality. Therefore, this research aimed to analyze the associations of childhood overweight with kidney cancer mortality and diabetes at the population level using multivariate methods.

## 2. Materials and Methods

### 2.1. Research Model

In this study, kidney cancer mortality, childhood overweight, and diabetes variables were defined in relation to healthcare expenditures, life expectancy, and mortality levels using a descriptive survey model. Subsequently, the associations of the research variables on kidney cancer were analyzed longitudinally using a correlational survey model. The study was modeled as longitudinal, retrospective, and prevalence-based.

### 2.2. Research Data

The study used data from the World Health Organization (WHO) and the World Bank for Türkiye between 2000 and 2023. Kidney cancer mortality and diabetes prevalence data were used from the WHO database. Health expenditure, life expectancy, mortality, and childhood mortality data were used from the World Bank. Since the study included all parameters for the relevant time period using a complete census sample, no power analysis was performed. Kidney cancer mortality data are given as figures by year, and these figures are correlated with population growth values to reflect aging levels in the population. Health expenditures are given as a percentage of GDP due to economic changes over the years. Childhood overweight is also given as a percentage among children. The distribution of the parameters used in the study was as follows:

WHO data: Kidney cancer mortality, diabetes prevalence

– Kidney cancer mortality (https://platform.who.int/mortality/themes/theme-details/topics/indicator-groups/indicator-group-details/MDB/kidney-cancer, accessed on 12 February 2026);
– Diabetes, prevalence (%) (https://www.who.int/data/gho/data/indicators/indicator-details/GHO/prevalence-of-diabetes-age-standardized, accessed on 12 February 2026).

World Bank data: Health expenditures, life expectancy

– Current health expenditure (% of GDP);
– Life expectancy at birth, total (years);
– Mortality from CVD, cancer, diabetes or CRD between exact ages 30 and 70 (%);
– Childhood overweight: Prevalence of overweight, weight for height (modeled estimate, % of children under 5);
– Population, total.

The kidney cancer mortality rate was calculated: as follows: Kidney cancer mortality rate = Kidney cancer deaths/total population (per year).

Kidney cancer mortality figures for each year were obtained from the number of deaths reported by health institutions and the WHO for that year. The study used life expectancy and health expenditure data from the World Bank as confounders. Additionally, the study compared results using a GLM that factored in the population growth ratio.

### 2.3. Ethical Approval and Informed Patient Consent

The study does not include personal information, and the patient numbers are prevalence-based. Therefore, informed patient consent or ethics committee approval is not applicable to this research. Since the data used are publicly available on the official websites of the WHO and the World Bank without requiring permission, institutional permission is not relevant. The Helsinki Declaration and Good Clinical Practice guidelines were followed during the writing process.

### 2.4. Statistical Methods

Scale parameters were described by the mean, standard deviation, median, and range of variation. Parity analysis was used in the time series description. Spearman’s rho correlation analysis was performed in the relational scanning analysis. In the association analysis, Generalized Linear Model (Logit) analysis was performed due to linearization deviations [18]. In the GLM analysis, kidney cancer mortality levels were used in a multivariate analysis with a new variable, proportionally adjusted for population growth rates, to demonstrate the association of population and age variables and to make it suitable for GLM analysis. The final dependent variable was defined as the annual kidney cancer incidence rate (total cases divided by the total population). Because this rate represents fractional data bounded between 0 and 1, a Generalized Linear Model (GLM) assuming a Binomial distribution with a Logit link function was estimated in IBM SPSS (25.0 for windows) Statistics. To address potential multicollinearity arising from strong temporal trends among population-level indicators (such as diabetes prevalence, life expectancy, and health expenditures), collinearity diagnostics were rigorously evaluated using the Variance Inflation Factor (VIF) before interpreting the regression coefficients. All analyses were performed using SPSS 25.0 and EViews 7.0 for Windows, with a 95% confidence interval and a significance level of 0.05. For the unit root test, Augmented Dickey–Fuller (ADF) and Phillips–Perron (PP) tests were used. Autoregressive Integrated Moving Average (ARIMA) modeling was employed to account for autocorrelation and non-stationarity. To ensure stationarity prior to regression analysis, non-stationary variables were transformed using the first-order differencing method (d = 1). Model order selection was based on the Bayesian Information Criterion (BIC), and the independence of residuals was verified using the Ljung–Box Q test.

## 3. Results

The mean kidney cancer mortality was 1045.07 ± 134.95, with a range of 784.00–1214.00. Diabetes prevalence was 12.55 ± 2.18% among adults, and the mean prevalence of childhood overweight was 8.61 ± 1.20% among children under 5 years of age. The mean disease mortality was 18.33 ± 1.74% among individuals aged 30–70 years, and the mean health expenditure was 4.65 ± 0.44% of GDP. Life expectancy ranged from 71.93 to 77.74 years, with a mean value of 75.16 ± 1.81 years. The ADF and PP test results showed that all parameters in the research had unit roots (*p* > 0.05) (Table 1).

Spearman’s rho correlation analysis results showed that kidney cancer mortality was significantly correlated with health expenditure (r = −0.614; *p* < 0.05) and life expectancy (r = 0.596; *p* < 0.05) (Table 2).

GLM diagnostics confirmed a robust and stable model structure. The Omnibus Test demonstrated overall model significance (*p* < 0.05). The Deviance Value/df ratio was (DV/df: 1.05), indicating a well-specified model without overdispersion. Despite the high likelihood of temporal correlation among macro-indicators, multicollinearity did not distort the estimates; all predictors exhibited a Variance Inflation Factor (VIF) below 3.4, satisfying standard diagnostic thresholds. Generalized linear model results showed that the associations of diabetes prevalence (B = 0.039; *p* < 0.01), health expenditure (B = −0.154; *p* < 0.01) and life expectancy (B = 0.061; *p* < 0.01) with kidney cancer mortality were statistically significant at the univariate level. However, associations of diabetes prevalence, childhood overweight and health expenditure on kidney cancer mortality at the multivariate level were statistically insignificant (*p* > 0.05) (Table 3).

ARIMA model results showed statistically insignificant associations at the multivariable level (in accordance with GLM (*p* > 0.05) (Table 4).

An increase in diabetes prevalence was positively and significantly associated with kidney cancer mortality at the univariate level, but this relationship showed an irregular trend, suggesting that some other confounders may be effective (Figure 1).

## 4. Discussion

This study aimed to conduct a population-level multivariate analysis of the associations between childhood overweight and kidney cancer mortality and diabetes using World Bank and WHO data for Türkiye. The results showed that while the prevalence of childhood overweight and diabetes was statistically significant in univariate analysis, these associations were not statistically significant in multivariate analysis.

Kidney cancer, which has significant mortality rates worldwide every year [2], is actually a type of cancer whose mortality levels can be reduced with current and new treatment options and good management of the prognosis. However, for this, it is necessary to understand the risk factors well. Common risk factors for kidney cancer in the literature include smoking, obesity, diabetes, and the sociodemographic characteristics of patients [4,5,6,7,8,9,10]. However, although risk factors are predominantly studied in studies on the prevalence and incidence of the disease, it can be stated that studies associating them with mortality are relatively few. Among these, obesity and diabetes are important factors that should be focused on, as they are closely related to metabolism and kidney function. However, we have not come across enough studies that examine these factors at the population level and through macro data in a multivariate manner.

Studies on kidney cancer and diabetes have reported diabetes as both a risk factor for kidney cancer and a risk factor for mortality. In their study, Bonilla-Sanchez et al. [10] reported diabetes as a significant risk factor affecting both kidney cancer and poor prognosis. Kim et al. [16] similarly reported the significant associations between diabetes and kidney function and kidney cancer. A similar situation exists for the relationship between obesity and kidney cancer. In their study, Pati et al. [19] reported a relationship between obesity and cancer types, including kidney cancer, in terms of causality and poor prognosis. Lim et al. [20] reported that hormonal imbalance, which is disrupted by obesity, affects cancer types, especially kidney cancer, through metabolic systems. Larson et al. [21], in their study examining the relationship between obesity, cancer, and physical activity, also reported kidney cancer among the cancer types associated with obesity. In their study, Preza-Fernandes et al. [22] reported the relationship between obesity and kidney cancer, specifically the relationship between perirenal adipose tissue and carcinoma progression. In their study, Arabi et al. [23] reported that hypertension and insulin resistance play a significant role in the relationship between obesity and kidney disease. In their study, Kreiner et al. [24] reported that weight loss and obesity treatment methods are closely related to kidney function and disease. Ghazy et al. [25] reported the relationship between the severity of obesity and the incidence of kidney disease, highlighting the significant role of obesity in kidney disease.

In addition to studies on the relationship between obesity and overweight and kidney function [26,27,28], the association of childhood overweight with kidney and other diseases is the underlying mechanism in this research [29,30]. However, our results indicate that a research design with more confounders is needed to investigate these relationships at the population level.

Although research clearly reveals the relationship and mechanism of action between obesity and kidney cancer mortality levels, we have not encountered any studies that examine this relationship using a multivariate model, a correlational survey model, and at the population level. While our results, based on univariate analysis, are parallel to studies in the literature, the multivariate analysis results indicate that this relationship may be more complex and that further research is needed at the population level and using macro data.

### 4.1. Study Limitations

The most significant limitation of this study is the exclusion of confounders, a common characteristic of all population-based prevalence studies. Many factors influence kidney cancer mortality rates, including disease duration, comorbidities, age, gender, chronic medication use, nutrition, and economic status. However, since our study focuses on macro-level prevalence, further prospective research would be beneficial in supporting these factors. Another major limitation of the study is that population-level associations cannot be extrapolated to individual patients.

Another significant limitation of the study is the time series, which is the vertical length of the dataset. Due to the communication protocol between the WHO and national databases, data arrive with a delay. Therefore, the most up-to-date data on kidney cancer mortality levels is from 2023. Changes since then may require further controlled studies to assess population-wide variations in mortality levels. However, since our data includes all kidney cancer cases for each year, it is possible to state that the level of generalizability is high. The multivariable model includes several predictors despite only approximately 24 annual observations. This may lead to overfitting, unstable estimates and insufficient statistical power. Since the research was conducted in Türkiye, ethnic differences should also be considered as a limitation in interpreting the results. Another conceptual limitation is that the contemporaneous prevalence of overweight among children under five is being related to adult kidney cancer mortality during the same calendar years. The additional references concerning childhood obesity and kidney involvement do not fully establish the epidemiological rationale for this contemporaneous ecological association.

### 4.2. Contributions of the Study to Literature

The most important contribution of the study to the literature is demonstrating that while childhood obesity and diabetes do have a univariate association with kidney cancer deaths, their overall association is not statistically significant. This is important both in terms of the findings and because it includes all cases from 2000 to 2023 for Türkiye, and it makes a significant contribution in terms of evidence-based analysis. The study also contributes a novel approach by considering the associations between diabetes and childhood obesity together.

## 5. Conclusions

According to our findings, while the prevalence of childhood overweight and diabetes was significant in univariate analysis, their associations were insignificant in multivariate analysis. Further studies and controlled randomized trials are needed to reduce kidney cancer mortality and provide public benefit. As there is no data linking childhood obesity to kidney cancer risk in the same individuals, this study may provide population-level insights for future research; however, it does not directly establish a link between childhood obesity and kidney cancer on an individual basis. There is a need for appropriately designed individual-level longitudinal studies before any causal or clinical implications can be drawn.

## Figures and Tables

**Figure 1:**
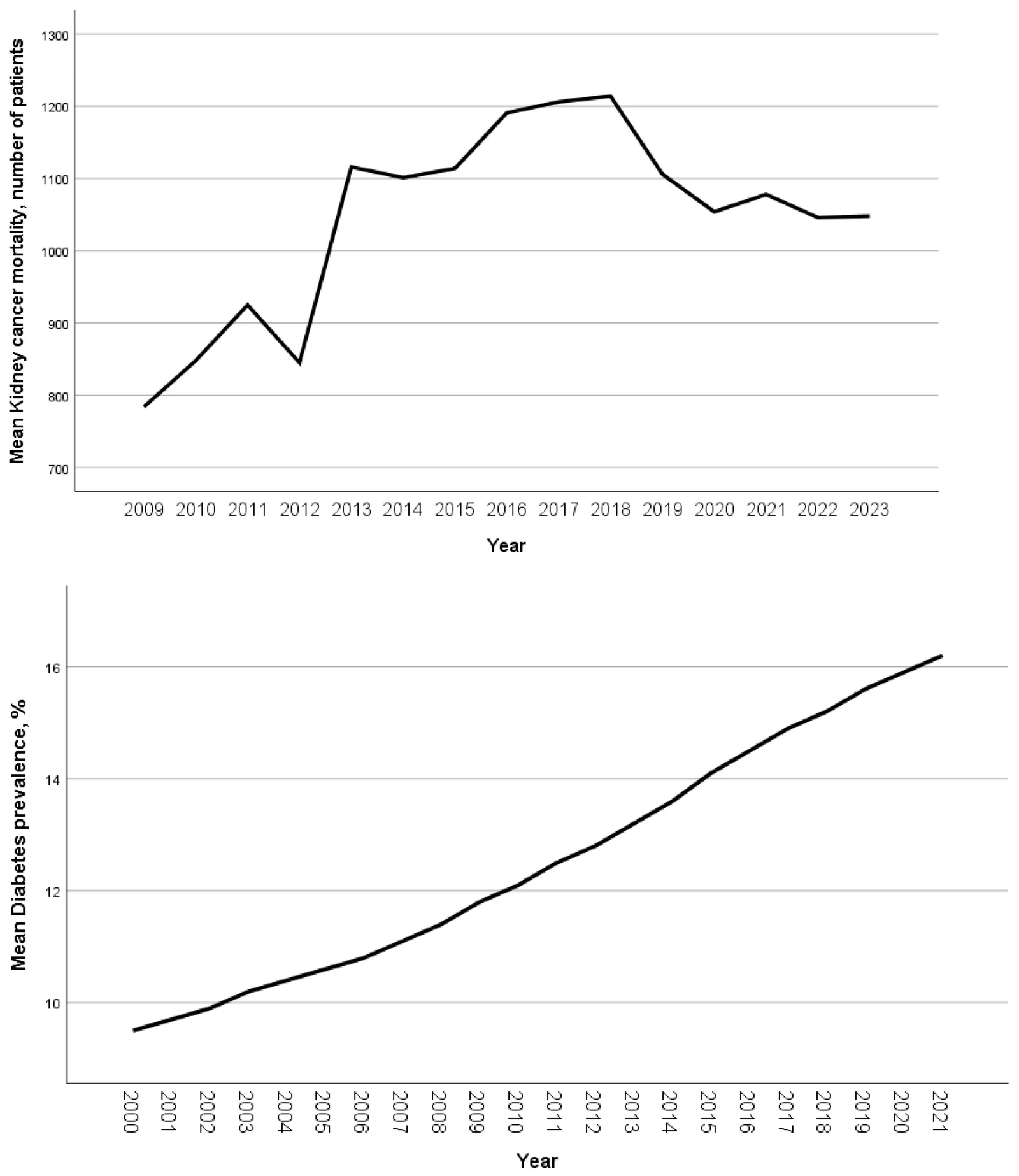
Change in diabetes prevalence and kidney cancer mortality for 2000–2023.

**Table 1:** Descriptive statistics for kidney cancer mortality, diabetes prevalence, childhood obesity, disease mortality, health expenditure and life expectancy.

	Mean	Standard Deviation	Minimum	Maximum	ADF	PP
Kidney cancer mortality ^a^	1045.07	134.95	784.00	1214.00	0.1984	0.1984
Diabetes prevalence ^b^	12.55	2.18	9.50	16.20	0.2590	0.9999
Childhood overweight ^c^	8.61	1.20	5.80	10.00	0.0004	0.1466
Disease mortality ^d^	18.33	1.74	15.20	22.50	0.1999	0.2445
Health expenditure ^e^	4.65	0.44	3.70	5.49	0.5187	0.5182
Life expectancy	75.16	1.81	71.93	77.74	0.2509	0.1948

^a^ patients, ^b^ %, ^c^ modeled estimate, % of children under 5, ^d^ mortality from CVD, cancer, diabetes or CRD between ages 30 and 70 (%), ^e^ % of GDP, ADF: Augmented Dickey–Fuller test, PP: Phillips–Perron Test.

**Table 2:** Spearman’s rho correlation between kidney cancer mortality, diabetes prevalence, childhood obesity, disease mortality, health expenditure and life expectancy.

Kidney cancer mortality ^a^	**r**	***p***
Diabetes prevalence ^b^	0.533	0.061
Childhood obesity ^c^	−0.256	0.357
Disease mortality ^d^	−0.523	0.067
Health expenditure ^e^	−0.614 *	0.015
Life expectancy ^f^	0.596 *	0.019

* *p* < 0.05. ^a^ patients, ^b^ %, ^c^ modeled estimate, % of children under 5, ^d^ mortality from CVD, cancer, diabetes or CRD between ages 30 and 70 (%), ^e^ % of GDP, ^f^ years.

**Table 3:** Generalized Linear Model (Logit) analysis for associations of diabetes prevalence, childhood obesity, disease mortality, health expenditure and life expectancy with kidney cancer mortality rate at univariate and multivariate level.

Parameter	B	Std. Error	95% Wald Confidence Interval		Hypothesis Test		
			Lower	Upper	Wald X^2^	df	*p*
***Multivariate level***							
Diabetes prevalence	0.045	0.106	−0.163	0.254	0.181	1	0.671
Overweight among children under 5 years	0.102	0.201	−0.293	0.496	0.256	1	0.613
Health expenditure	−0.103	0.158	−0.413	0.207	0.424	1	0.515
Life expectancy	0.044	0.033	−0.022	0.109	1.702	1	0.192
***Univariate level***							
Diabetes prevalence	0.039	0.018	0.004	0.075	4.626	1	0.032
Overweight among children under 5 years	−0.005	0.035	−0.074	0.065	0.018	1	0.893
Health expenditure	−0.154	0.055	−0.261	−0.047	7.953	1	0.005
Life expectancy	0.061	0.023	0.016	0.107	6.975	1	0.008

**Table 4:** ARIMA Model analysis for associations of diabetes prevalence, childhood obesity, disease mortality, health expenditure and life expectancy on kidney cancer mortality at the univariate and multivariate level.

	Coefficient	Std. Error	t-Statistic	Prob.
C(1)	−3624.668	6186.605	−0.585890	0.5741
C(2)	56.81903	137.0996	0.414436	0.6894
C(3)	65.19061	259.2017	0.251505	0.8078
C(4)	−120.3469	203.8624	−0.590334	0.5713
C(5)	49.96445	43.07006	1.160074	0.2795
R-squared	0.827026	Mean dependent var		1044.769
Adjusted R-squared	0.740540	S.D. dependent var		145.7613
S.E. of regression	74.24681	Akaike info criterion		11.73639
Sum squared resid	44,100.71	Schwarz criterion		11.95368
Log likelihood	−71.28653	Hannan–Quinn criter.		11.69173
F-statistic	9.562458	Durbin–Watson stat		2.290270
Prob(F-statistic)	0.003857			

KIDNEY_CANCER = C(1) + C(2) × DIABETES PREVALENCE + C(3) × OBESITY + C(4) × HEALTH_EXPENDITURE + C(5) × LIFE_EXPECTANCY.

## Data Availability

The data that support the findings of this study are publicly available from World Health Organization and World Bank Country Reports on their official websites.

## Abbreviations

The following abbreviations are used in this manuscript:

GDP	Gross Domestic Product
WHO	World Health Organization
